# GAAOA-Lévy: a hybrid metaheuristic for optimized multilevel thresholding in image segmentation

**DOI:** 10.1038/s41598-025-12142-z

**Published:** 2025-07-26

**Authors:** Eman Mahmoud, Salem Alkhalaf, Tomonobu Senjyu, Masahiro Furukakoi, Ashraf Hemeida, Ghada Abozaid

**Affiliations:** 1 Faculty of Science, Aswan University, Aswân, 81528 Egypt; 2 Department of Computer Engineering, College of Computer, Qassim University, Buraydah, Saudi Arabia; 3 Department of Electrical and Electronics Engineering, University of the Ryukyus, Okinawa, 903-0213 Japan; 4 Faculty of Engineering, 1-1-1 Daigaku-Dori, Sanyo Onoda, Yamaguchi 756-0884 Japan; 5 Faculty of Energy Engineering, Aswan University, Aswan, 81528 Egypt; 6 Faculty of Engineering, Aswan University, Aswan, 81528 Egypt

**Keywords:** Image segmentation, Genetic algorithm (GA), Archimedes optimization algorithm (AOA), Multilevel thresholding, Electrical and electronic engineering, Computational science

## Abstract

Image segmentation is a critical task in image processing with applications in various domains, including industry and medicine. However, multilevel thresholding, a widely used segmentation technique, suffers from high computational complexity due to the exhaustive search for optimal thresholds. This paper addresses this challenge by proposing a hybrid Genetic Algorithm-Archimedes Optimization Algorithm (GAAOA), further enhanced with a Lévy flight function (GAAOA-Lévy), to improve efficiency and accuracy in multilevel thresholding. The integration of GA’s crossover mechanism strengthens local search capabilities, leading to optimal segmentation with fewer iterations. The proposed algorithm is evaluated using standard benchmark images and compared against well-known optimization techniques. Experimental results demonstrate that GAAOA-Lévy outperforms existing methods in terms of Peak Signal-to-Noise Ratio (PSNR), computational efficiency, and convergence speed, particularly excelling in three-level thresholding while reducing computational costs for higher thresholds.

## Introduction

Image processing has recently found use in a variety of fields, including industry, agriculture, and medicine. The most crucial stage of image processing is thought to be image segmentation^1^. According to the intensity magnitude of the pixel, image segmentation categorizes the pixel in an image. Different approaches have been put forth in the literature for segmenting images, such as edge-based techniques^2^, neural network techniques^3^, watershed techniques^4^, clustering techniques^5^, and artificial threshold techniques^6^.

The threshold approach is a quick and efficient way to separate interesting elements from the background. It is used for a number of traditional tasks, for instance, document image analysis, which seeks to detect lines, legends, and letters^7^, as well as map processing, which seeks to locate logos, graphical information, or musical scores. In order to detect and mark objects, the threshold approach is also employed in scene processing^8^. Similarly, it has been used to conduct quality checks on materials before tossing out faulty components^9^. The image is divided into couple (bi-level) or extra (RGB) using thresholding algorithms. Using only one threshold value (t), the binary level thresholding tests each pixel’s intensity magnitude and, if it is maximum, classifies the threshold magnitude (t) as the first category and the other pixel’s intensity value as the second class. The pixels in the image are separated into multiple classes during multilayer thresholding, and each class is given a different threshold magnitude^10–12^. To find the ideal threshold magnitude, one can essentially choose between parametric and nonparametric methods^11^. For the purpose of classifying the various image classes using a parametric technique, some probability density function parameters should be computed. However, compared to the nonparametric method, this approach requires more time and more processing resources to optimize a number of factors, including the deviation rate and entropy, to obtain the accurate threshold values. However, Otsu’s and Kapur’s approaches were employed for binary level thresholding. The Otsu technique maximizes the variation between classes^13^. To assess the homogeneity of the classes, the Kapur technique maximizes entropy^14^. With each subsequent threshold, the two multilayer thresholding algorithms’ computing complexity rises^15^. Whale Optimization Algorithm (WOA)^16^, Ant Colony Optimization (ACO)^17^, Particle Swarm Optimization (PSO)^18^, Moth Flame Optimization (MFO)^16^, Genetic Algorithm (GA)^19^, and Moth Flame Optimization (MFO) are just a few optimization algorithms that segment images with multilayer thresholding. The Ensemble Genetic Algorithm Explainer (EGAE), which recognizes and displays to the viewer the informative portions of the image automatically for the identification of melanoma cancer, has been discussed and investigated^20^. Three phases make up EGAE. First, a heuristic method is used to determine the hromosomal sparsity in GAs. Then, several GAs are run one after the other. However, these GAs differ from one another in that the input image contains a varied amount of super-pixels, which causes the chromosomal lengths to vary. Finally, majority and consensus voting are used to combine the outcomes of GAs. According to experimental findings on a melanoma dataset, EGAE efficiently enhances explanation accuracy when compared to LIME and discovers informative lesions automatically.

Multilevel genetic algorithms-based segmentation approaches for medical images were suggested in^21^. While the Quantum Genetic Algorithm (QGA) uses the qubit encoding of people, the GA uses binary coding. To efficiently optimize Rényi, Masi, and Shannon entropies for the purpose of segmenting multiple objects in medical imaging, two evolutionary techniques are used. For comparison, the PSO was also implemented^21^. The three most widely used segmentation indices, PSNR, Structural Similarity Index Measure (SSIM), and FSIM, were used to evaluate the segmentation accuracy of the nine proposed methodologies. Twenty medical images were chosen as a sample for the numerical results and comparison investigation. Finally, it was discovered that the Rényi entropy is better suited for multilevel thresholding in medical images^21^. Its purpose was to assess the growth of small enterprises in the Russian Far Eastern District with respect to sustainability’s environmental, social, and economic dimensions^22^. International regulators expect national governments to establish a digital asset regulatory framework akin to that which is now in place for traditional finance^23^. To look into the connection between the cost and demand of Bored Ape NFT assets, data from OpenSea, SuperRare, and Nifty Gateway were used. The cross-quantilogram method is used to examine the relationship between the Bored Ape NFT Collection’s popularity and price^24^. Another study^25^ looked at South Korea’s approach to the world oil market.

Genetic crossover operation and smart inertia weight (SGA-BA) were suggested to select the best thresholds^26^. Additionally, the Otsu method’s between-class variance and Kapur’s entropy are used as objective functions. According to the number of iterations and fitness values, the smart inertia weight in the innovative SGA-BA balances the SGA-BA’s exploration and exploitation. Furthermore, the crossover operation of the genetic algorithm strengthens the capacity to search locally the SGA-BA. The beta distribution takes the place of the random vector in the interim, smartly updating the frequency of bats. An assortment of benchmark photos with varied threshold levels was used to assess the proposed SGA-BA. PSO, GA, GSA, WOA, and LSSA were all put up against each other. The experimental findings demonstrate that the suggested SGA-BA offers superior results to the other algorithms^26^.

The automatic clustering of unlabeled pixels from MR images into various homogenous groups using a segmentation approach based on a GA was investigated^27^. This approach does not call for knowledge of the ideal number of segments or the underlying pixel distribution prior to segmentation. The fuzzy inter-cluster hostility index classifies the centroid of various segments as active or passive. The images used as a test are then divided up by the chosen active centroids. This method yields the accurate number of segments and their corresponding centroids. With the aid of two real-world MR images, a behavior comparison is shown between the fuzzy inter-cluster hostility index-based GA approach, the renowned automated clustering utilizing differential evolution (ACDE) methodology, and one non-automatic algorithm.

Histogram-based methods lack the spatial intricacies of contextual information necessary to determine the ideal threshold values. To address this, a unique approach called the Energy Curve is proposed, which uses Otsu’s method and the Harmony Search Algorithm to compute optimum gray levels instead of a histogram. The suggested approach was tested on a number of benchmark photos, and the outcomes were compared using a histogram and several optimization algorithms. The comparison of the SD Index, mean of fitness, and PSNR makes it clear that the suggested approach outperforms histogram-based techniques^28^. In medical image analysis, picture segmentation is essential, especially for precisely identifying tumors and lesions. Efficient segmentation enhances the accuracy of diagnosis and streamlines quantitative analysis^29^. However, multilevel thresholding presents a challenge to typical segmentation techniques because of the resulting computing complexity. As a result, choosing the ideal threshold set is an NP-hard task, underscoring the urgent need for effective optimization techniques to get beyond these obstacles^29^. We explored and studied HADECO, a hybrid strategy combining Differential Evolution (DE) and the Crayfish Optimization Algorithm (COA) for multi-threshold image segmentation (MTIS). By employing a two-dimensional (2D) histogram and 2D Kapur’s entropy, this technique seeks to improve the effectiveness and precision of subsequent picture processing and diagnosis^29^. The Harris Hawks Optimizer (HHO) and Archimedes Optimization Algorithm (AOA) were combined to enhance AOA during the exploitation phase and produce the ideal threshold vector for MTIS. As a result, the hybrid AOA-HHO algorithm outperforms both AOA and HHO algorithms as well as a few other MH algorithms in solving the MTIS problem. It also achieves better thresholds than AOA and HHO, which improve the MTIS system’s performance. HHO is powerful during the exploitation phase, and AOA is powerful during the exploration phase^3^.

In terms of image segmentation accuracy, fitness function value, PSNR, SSIM, and execution time, experiments demonstrate that the AOA-HHO technique outperforms the other algorithms and even HHO and AOA^30^. The direct and useful application of MTIS in medical image segmentation is well known. To improve image segmentation accuracy, an improved version of the Whale Optimization Algorithm (CVWOA) was created. By combining vortex rotation and convex local directed search, the CVWOA greatly enhances the algorithm’s overall optimization performance. This technique performed segmentation on the Berkeley Segmentation using non-local means 2D histograms and Renyi’s entropy^31^. It was created to increase cuckoo search’s (CS) efficacy. Enhancing exploration, exploiting possibly novel solutions, and preventing local optima are the objectives. In order to increase the core CS framework’s exploration and exploitation capabilities, we introduce three new algorithms—the grey wolf optimizer (GWO), red panda optimization (RPO), and naked mole rat algorithm (NMRA)^32^. The selection of a threshold is a major issue as the number of threshold segmentations increases, so an optimization approach for random collision whales was proposed to optimize OTSU for dependable picture segmentation^33^. Using CNNs and vision transformers, a novel deep learning architecture was shown for medical image segmentation. Using biconvolutional long-short-term memory (LSTM) networks and vision transformers (ViT), this model, called TBConvL-Net, is a hybrid network that combines the local properties of a CNN encoder–decoder architecture with long-range and temporal dependencies^34^. A review of current picture segmentation techniques that have undergone thorough algorithmic categorization was given. Several assessment criteria were examined in order to compare the outcomes of various segmentation methods. Additionally, a thorough explanation of the various application domains for picture segmentation was given^35^.

The comparison showed that the GA automatic image segmentation approach utilizing the fuzzy inter-cluster hostility index was superior to the other two algorithms^4^. To identify huge tumor sizes for diagnostic and therapy planning, medical picture segmentation is often done manually by a doctor^36^. Medical professionals manually segment organs using their prior knowledge of their locations and forms; however, this method is vulnerable to reader subjectivity and inconsistent results. Due to the poor tissue contrast and hazy organ/tissue borders in medical pictures, automating the process is difficult. In order to accomplish automatic three-dimensional segmentation, this research introduces a genetic method for merging illustrations of learnt information, like recognized forms, regional attributes, and the objects’ relative positions within a unified framework. Both magnetic resonance imaging and pelvic computed tomography have been used to test the algorithm’s prostate segmentation capabilities^36^.

A hybrid GAAOA technique is suggested for use in multilayer thresholding for image segmentation in this paper. The suggested method is validated using two standard images and compared to other renowned optimization methods such as electromagnetic optimization-based Lévy function (EMO Lévy), EMO, CS, Sine Cosine Algorithm (SCA), MFO, and WOA. The results show that the suggested algorithm produces better multilevel segmentation of digital images in comparison with other known algorithms, with fewer iterations and quick calculation times.

The document is structured in the manner described below. The hybrid GAAOA is illustrated in Section “Hybrid GAAOA”. The implementation of the hybrid GAAOA algorithm for developing the image segmentation-based multilevel thresholding problem is shown in Section “Image segmentation using GAAOA”. Section “Results and discussions” provides the simulation results and discussions and compares the created algorithm’s comprehensive results with those from other approaches. The advantages and drawbacks of the suggested algorithm are provided in Section “Advantages and drawbacks of the suggested hybrid GAAOA”. Finally, Section “Conclusions” illustrates the paper’s conclusions.

## Hybrid GAAOA

### Genetic algorithm (GA)

**Fig. 1:**
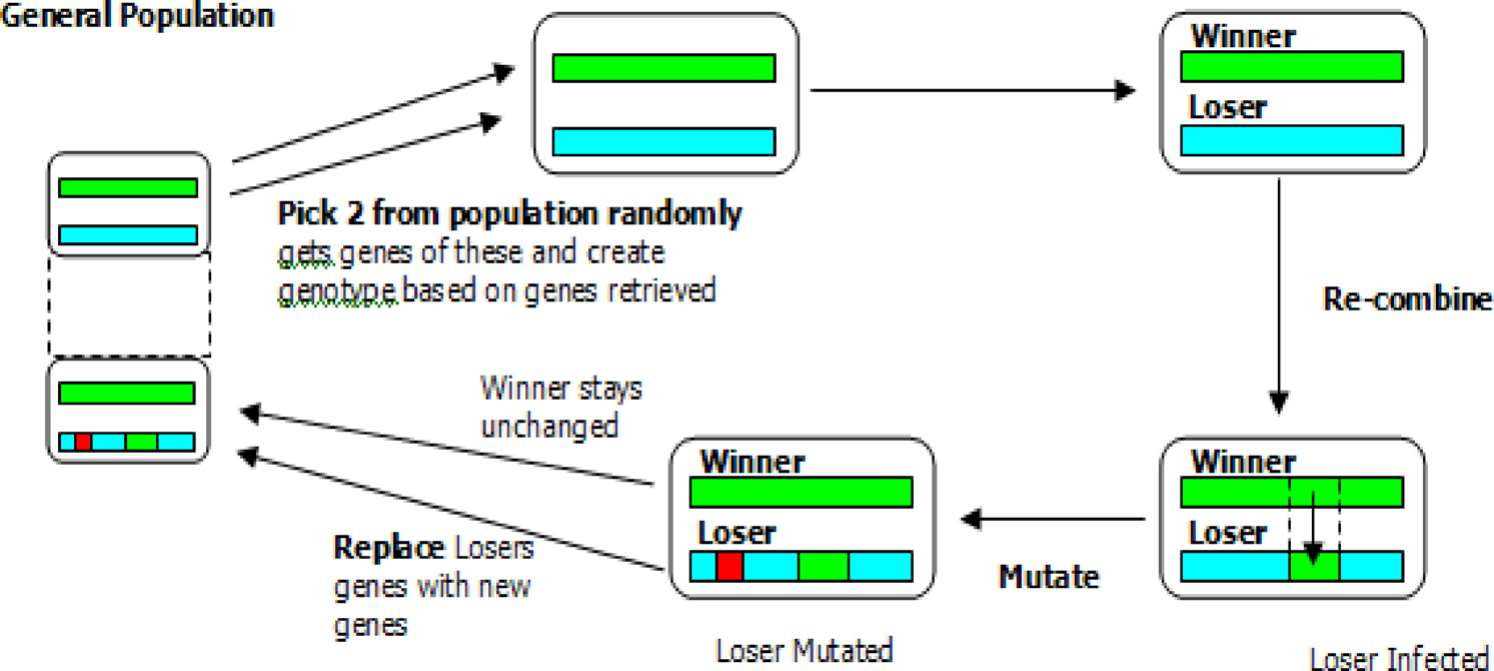
Genetic algorithm technique ^37^.

GA is a search technique used by computers to locate precise or actual response to search and optimization issues. Worldwide search algorithms are a category that includes GAs. There are various evolutionary algorithms that use mechanisms evolutionary biology serves as a model for concepts like heredity, mutation, selection, and crossover. Figure 1 depicts the GA method.

**Fig. 2:**
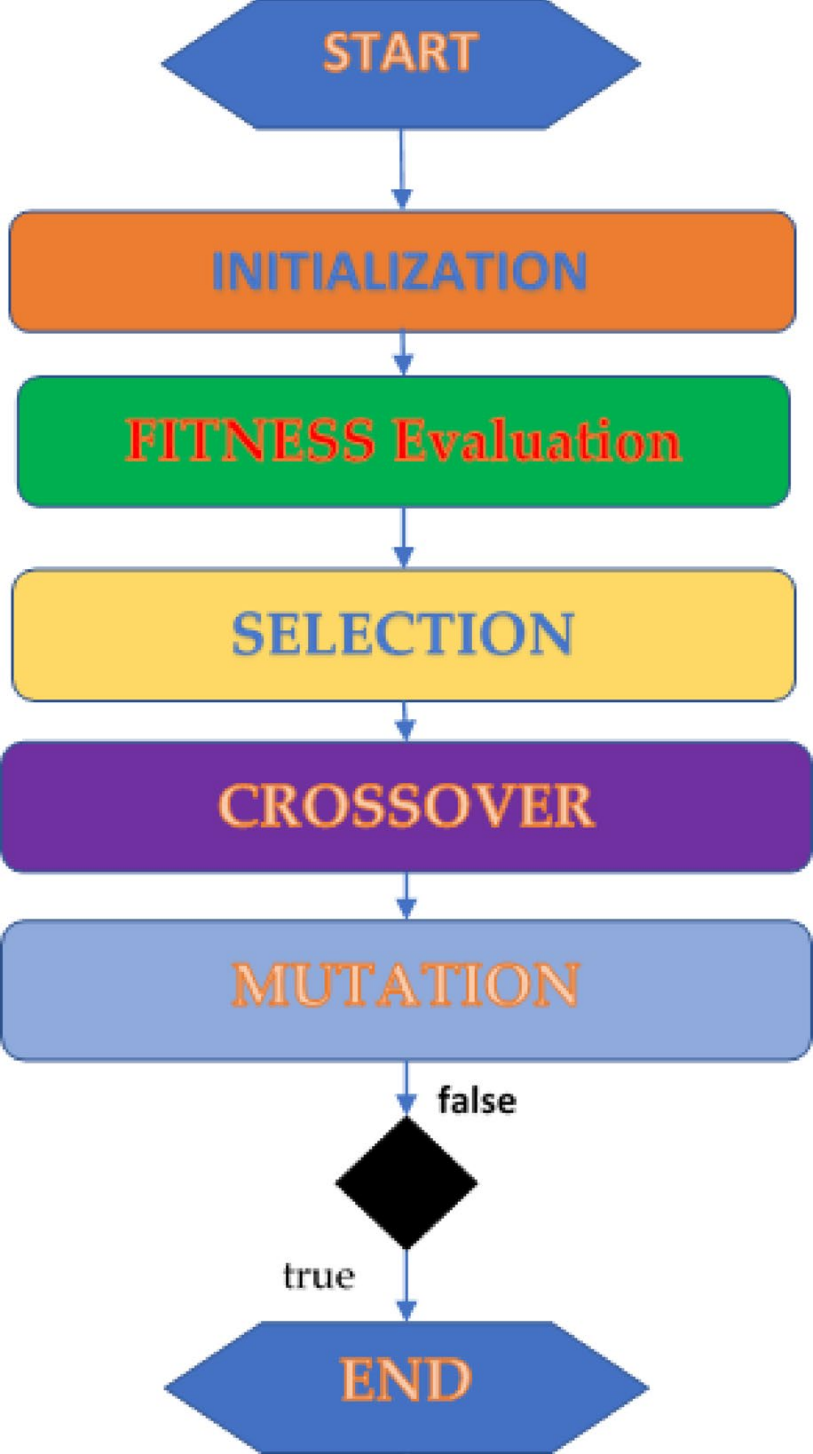
Flowchart for a genetic algorithm ^37^.

In a simulation using genetic approaches, a population of abstract models for potential solutions to an optimization problem—referred to as phenotypes, animals, or people—becomes closer to superior answers—referred to as chromosomes, genotypes. Typically, solutions are stated as binary strings of 0s and 1s; however, other encodings are also possible. Generational evolution starts primarily with a population that is initialized at random. Every generation, the fitness of each group member is determined. To create a new population, a number of individuals are chosen at random from the existing population (based on their fitness magnitude) and altered (recombined, or even mutated). The next iteration of the algorithm then makes use of the new population^38–40^. The flow chart for the GA is shown in Fig. 2.

#### Solution steps of GA

*Step one* (Initialization): Put t = 0 in the time counter.

*Step two*: Create a population of n response or chromosomes at random.

*Step three* (Evaluation): For each chromosome that is a section of the population, compute the fitness function. The major population’s chromosomes are examined to determine which one is best. The best chromosome among them all is Xbest, which generates the best objective function.

*Step four* (Keep track of the time): As time passes, it gets longer (t = t + 1).

*Step five*: a new population is added and created in accordance with the following rules.

Based on their potential to produce accurate response for the following generation, choose a few solutions.

Crossover: To produce new offspring, the parents are crossed.

Mutation: Depending on the capacity for mutation, the new chromosomes may change.

Acceptance: Transfer of the new generation of the new populace.

*Step six*: Replace the freshly created solutions with ones that were chosen at random in step five.

*Step seven*: If a termination condition is met, quit; otherwise, go back to step two.

### Archimedes optimization algorithm (AOA)

**Fig. 3:**
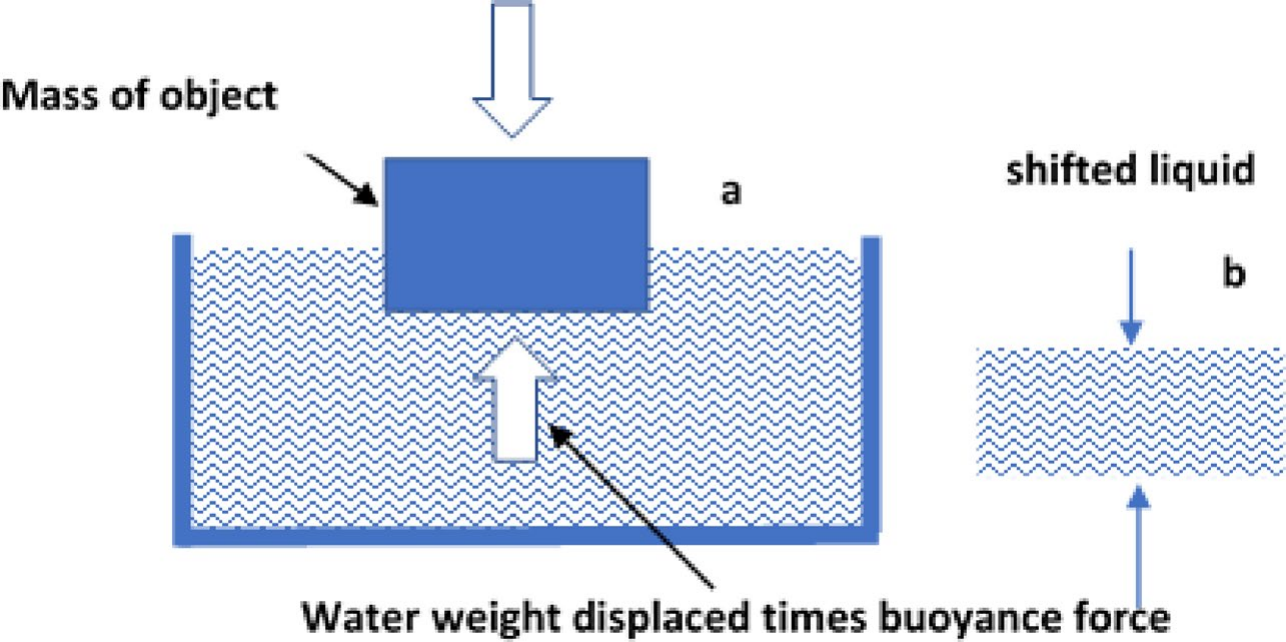
(**a**) object submerged in fluid, and (**b**) The volume of fluid expended ^37^.

AOA stands out for being simple to employ and requiring fewer regulating factors (population size and ending criteria)^41^. The essential statements of Archimedes serve as the foundation for this optimizer. AOA describes the behavior when it is just partially or fully immersed in a fluid, with the fluid exerting pressure on an upward force on the object proportionate to the displacement the object causes with respect to the fluid. A buoyant force, which is equivalent to the weight of the displaced fluids, is applied to an object when it is submerged in a liquid (see Fig. 3)^42^.

Pressure vessel design, speed reducer design difficulties, and welded beam architecture have all been addressed successfully using the AOA^43^. It has the potential to handle complex optimization issues while offering faster, more accurate global optimization. However, there is a lack of research on how AOA^44^ handles difficulties with DG creation and network resiliency. AOA is a population-based metaheuristic method making use of a population of things (possible solutions) with a range of volumes, accelerations, and densities. It initiates the search at random. Each object is now dispersed randomly across the fluid. The optimization procedure continues until the termination condition is satisfied or until all iterations have been completed. Each iteration alters the density and volume of every object. Whether or not an object’s acceleration changes depends on its propensity to collide with nearby objects. The updated location of an item will take new density, volume, and acceleration into account. Below is a comprehensive mathematical simulation of the AOA stages.

#### AOA solution steps

Initialization:

Set the algorithmic parameters C1, C2, C3, C4, u, and I, where C1, C2, C3, and C4 are constants with values of 2, 6, 2, and 0.5, respectively. The normalization’s maximum and minimum bounds (u and I) are changed to 0.9 and 0.1, respectively.

1 $$X_{i} = lb_{i} + rand{ }x{ }\left( {ub_{i} - lb_{i} } \right);i = 1,2, \ldots ,N$$

N is the upper limit number of objects, i is the number of objects, and x indicates the item. The bottom and top boundaries of the search space are denoted by lbi and ubi, respectively. Set the volume (vol), acceleration (acc), and density (den) initial values for each ith number.

2 $$den_{i} = rand$$

3 $$acc_{i} = lb_{i} + rand{ }x{ }\left( {ub_{i} - lb_{i} } \right)$$

4 $$vol_{i} = rand$$

rand is a vector of randomly generated values in the range [0, 1].

The fitness value can be calculated from the following:

5 $$Y_{i} = fobj{ }\left( {X_{i} } \right)$$

To select the best item in terms of fitness value, fobj is a function that computes first population estimate. Xbest, denbest, accbest, and volbest should be assigned, with denbest, accbest, and volbest. The best object yet discovered is connected with the volume, acceleration, and density.

The transfer operator is calculated by

6 $$TF = {\text{exp}}\left( {\frac{{t - t_{max} }}{{t_{max} }}} \right).$$

The density factor is calculated by.

7 $$d^{t + 1} = {\text{exp}}\left( {\frac{{t - t_{max} }}{{t_{max} }}} \right) - \left( {\frac{t}{{t_{max} }}} \right).$$

The following equations update the density and volume:

8 $$den_{i}^{t + 1} = den_{i}^{t} + rand{ }x{ }(den_{best} - den_{i}^{t} ).$$

9 $$vol_{i}^{t + 1} = vol_{i}^{t} + rand{ }x{ }(vol_{best} - vol_{i}^{t} ).$$

If TF is less than 0.5, an object collides with another. Use the following equation to modify the acceleration of a random-material object (mr) for iteration t + 1:

10 $$acc_{i}^{t + 1} \frac{{den_{mr} + vol_{mr} { }x{ }acc_{mr} }}{{den_{i}^{t + 1} { }x{ }vol_{i}^{t + 1} }}$$

Phase of exploitation (no collision):

If TF > 0.5, use the following formula to update object acceleration for iteration t + 1:

11 $$acc_{i}^{t + 1} = \frac{{den_{best} + vol_{best} { }x{ }acc_{best} }}{{den_{i}^{t + 1} x{ }vol_{i}^{t + 1} }}{ }$$

Normalize acceleration.

The formula used to determine the percentage change is

12 $$acc_{i,norm}^{t + 1} = u{ }x{ }\frac{{acc_{i}^{t + 1} - {\text{min}}\left( {acc} \right)}}{{\max \left( {acc} \right) - {\text{min}}\left( {acc} \right)}} + I$$

Each agent’s step will vary based on the percentage of ($acc_{i,norm}^{t + 1}$). For objects outside of the ideal global zone, the acceleration value is high meaning. In the same way, the search changes from discovery to application.

Update position.

The ith item location for iteration t + 1 applies Eq. (13) if TF 0.5 (exploration phase):

13 $$X_{i}^{t + 1} = X_{i}^{t} + C_{{1{ }}} x{ }rand{ }x{ }acc_{i,norm}^{t + 1} { }x{ }d{ }x(X_{rand} - X_{i}^{t} ),$$

Otherwise, the objects update their positions using Eq. (14).

If TF > 0.5 (exploitation phase),

14 $$X_{i}^{t + 1} = X_{best}^{t} + F{ }x{ }C_{{2{ }}} x{ }rand{ }x{ }acc_{i,norm}^{t + 1} { }x{ }d{ }x\left( {TX_{best} - X_{i}^{t} } \right)$$

15 $${\text{T}} = C_{3} x TF$$

where F is changing the direction of motion with the flag,

16 $$F = \left\{ {\begin{array}{*{20}l} { + 1} \hfill & {if} \hfill & {P \le 0.5} \hfill \\ { - 1} \hfill & {if} \hfill & {P > 0.5} \hfill \\ \end{array} } \right.$$

17 $${\text{ P = 2}} \times {\text{rand}} - C_{4}$$

Evaluation:

Consider each object in light of objective function (f), and then keep the top result thus far. Determine the following parameters’ values:${ }X_{best} ,den_{best} { },vol_{best} { }and{ }acc_{best}$

### Hybrid GAAOA technique

**Fig. 4:**
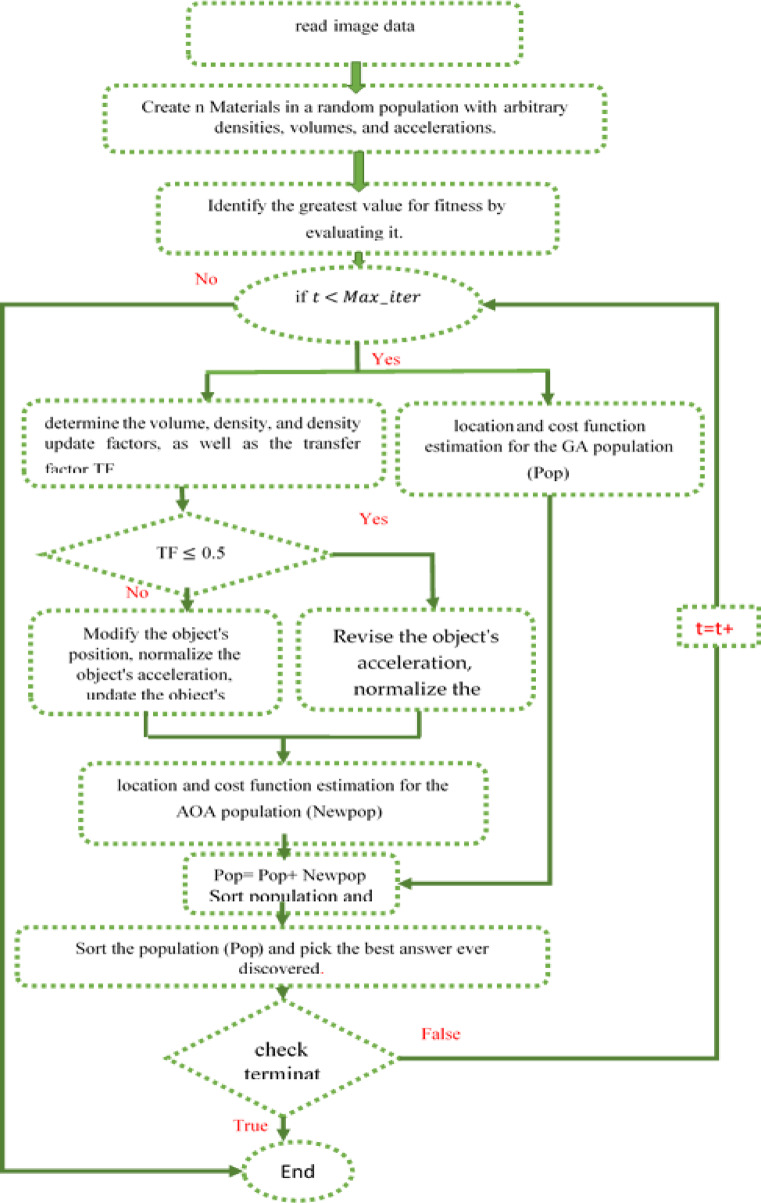
GAAOA Flowchart.

The flowchart discussing the implementation of hybrid GAAOA algorithm is depicted in Fig. 4. As was already said, GA has a number of drawbacks, like the possibility that can be obtained through many generations to get the ideal. As a result, it is quite expensive and takes a long time to compute the performance overall and product quality improved in order to overcome this limitation and lessen the amount of time required to analyze a function. A hybrid approach has been suggested. When utilizing programming languages like C++, a hybrid algorithm selects one (depending on the input) or switches between them throughout the process, mixing different algorithms to address the same problem. This is typically done to combine the beneficial aspects of each component so that the overall strategy performs better than the sum of the parts.

In this article, a novel hybrid method that combines the Archimedes Optimization method with the Genetic Algorithm is proposed, that is, GAAOA. The suggested approach has a number of distinctive qualities. First, because AOA can be faster and provide accurate solution compared to GA, it is used to avoid the local minimum solution. Because AOA can quickly determine the appropriate DG size and position, it has a considerable effect on the search procedures. Second, the algorithm’s performance is improved by dividing the optimization population. The GA algorithm is applied on the initialized population’s first half, and each time the GA operators make a decision, this half of the population is steadily improved.

## Image segmentation using GAAOA

Thresholding is an efficient technique for segmenting images. According to the parameters of the image’s intensity level (L), this approach is utilized to categorize binary level images and multilevel images. The image is changed into (mn) pixels throughout this process. Each pixel carries a value for its intensity level (L), which is categorized according to the class to which it belongs.

In case of greyscale image, the thresholding technique divides it into couple classes, R1 and R2, using simply a threshold value (th). As a result, if a pixel’s intensity level parameter is greater than th, it can be placed in class R1; otherwise, it will be placed in class R2.

18 $$\begin{gathered} R_{1} \leftarrow p if 0 < p < th \hfill \\ R_{2} \leftarrow p if th < p < L - 1 \hfill \\ \end{gathered}$$

Since there are more than two classes in a multilayer image, each class having its own threshold value, the classes in the multilayer image ${\text{TH}} = \left( {{\text{th1}},{\text{th2}},{\text{th3}}, \ldots {\text{th}}\;{\text{L}} - {1}} \right)\;{\text{are}}\;\left( {{\text{R1}},{\text{R2}}, \ldots {\text{RN}}} \right)$ N is the quantity of classes.

19 $$\begin{array}{*{20}l} {R_{1} \leftarrow p} \hfill & {if} \hfill & {0 < p < th} \hfill \\ {R_{2} \leftarrow p} \hfill & {if} \hfill & {p > th_{1} } \hfill \\ {R_{3} \leftarrow p} \hfill & {if} \hfill & {p > th_{2} } \hfill \\ {R_{N} \leftarrow p} \hfill & {if} \hfill & {p > th_{N - 1} } \hfill \\ \end{array}$$

The challenge with thresholding is choosing the th parameters that accurately categorize the classes for bi-level and multi-level thresholding. The methods of Otsu and Kapur are well-known techniques for calculating such values. For calculating the best threshold response, the goal functions proposed by the two methods must be maximized, as detailed below.

### Otsu’s technique

One technique for segmenting an image is the Otsu approach, which maximizes variance between classes before determining the magnitude of the objective function as follows:

20 $$f (th)_{otsu } = {\text{max}}\left( {{\upsigma }_{b}^{{2^{r} }} \left( {{\text{th}}} \right)} \right)\;where\;0 \le {\text{th}} \le {\text{L}} - 1{ }{\text{.}}$$

To achieve a greater threshold intensity level that increases (20), the optimization issue is reduced. Because it has one threshold (th), the prior aim is utilized for grey rate images. To apply to RGB pictures, Eq. (20) can be modified as follows:

21 $$f (TH)_{otsu } = {\text{max}}\left( {{\upsigma }_{b}^{{2^{r} }} \left( {{\text{TH}}} \right)} \right)$$

where $$0 < th_{i} < {\text{L}} - 1 = 1, \ldots ,{\text{k}}$$

TH = ($th_{1} ,th_{2} , \ldots th_{k - 1}$) and k is number of class

22 $$\sigma_{B}^{{2^{r} }} = \mathop \sum \limits_{i = 1}^{k} \sigma_{i}^{r}$$

23 $$\sigma_{i}^{r} = w_{i}^{r} (m_{i}^{r} - m_{t}^{r} )^{2}$$

where, i → identifies a specific class, and k is the number of classes, r → refers to a fixed value in a greyscale image that is equal to 1. (r = 1,2,3 in RGB image), ${\sigma }_{i}^{r}$→ refers to the variation between classes R (also known as Ottu’s variance),$m_{i}^{r}$→ refers to a class’s mean.

24 $$\begin{aligned} m_{0}^{r} & = \mathop \sum \limits_{i = 1}^{{th_{1} }} \frac{{iph_{i}^{r} }}{{w_{0}^{r} \left( {th_{1} } \right)}} \\ m_{1}^{r} & = \mathop \sum \limits_{{i = th_{1} + 1}}^{{th_{2} }} \frac{{iph_{i}^{r} }}{{w_{1}^{r} \left( {th_{2} } \right)}} \\ m_{k - 1}^{r} & = \mathop \sum \limits_{{i = th_{k} + 1}}^{L} \frac{{iph_{i}^{r} }}{{w_{1}^{r} \left( {th_{k} } \right)}} \\ \end{aligned}$$

where $w_{1}^{r}$→ refers to the likelihood of something happening,

$$w_{1}^{r} \left( {th} \right) = \mathop \sum \limits_{i = 1}^{{th_{1} }} ph_{i}^{r}$$

25 $$w_{2 }^{r} \left( {th} \right) = \mathop \sum \limits_{{i = th_{1} + 1}}^{{th_{2} }} ph_{i}^{r}$$

$$w_{k - 1}^{r} \left( {th} \right) = \mathop \sum \limits_{{i = th_{k} + 1}}^{L} ph_{i}^{r}$$

$ph_{i}^{r}$ is the probability distribution, which may be calculated as

26 $$ph_{i}^{r} = \frac{{h_{i}^{r} }}{N},r = \left\{ {\begin{array}{*{20}l} 1 \hfill & {in} \hfill & {grey\;image} \hfill \\ {1,2,3} \hfill & {in} \hfill & {RGB\;image} \hfill \\ \end{array} } \right.$$

27 $$\mathop \sum \limits_{i = 1}^{N} ph_{i}^{r} = 1$$

The number of pixels that correspond to the i intensity level are shown by the histogram distribution values $h_{i}^{r}$ where N represents all of the image’s pixels. The segmentation problem’s threshold is utilized in electromagnetic optimization to choose the best decision variable, which can be found as follows:

Maximize $$f{ }\left( {TH} \right)_{ostu}$$

Subject to TH $\in$ x, ${\text{TH}} = (th_{1} ,th_{2} , \ldots th_{k}$)

0 < $th_{i}$. <255; this is minimum and maximum bounded of threshold i = ${1}, \ldots {\text{k}}$ and k is the different thresholds.

### Kapur technique

Kapur^42^ has suggested another nonparametric approach for identifying the ideal threshold values. It is fnded on the probability distribution and entropy of the image histogram. This approach seeks to identify the ideal *th* that maximizes total entropy. The compactness and class separability of a picture are measured by its entropy. Entropy is at its highest level in this meaning when the optimal *th* value adequately divides the classes. The objective function of Kapur’s issue for the bi-level example is

28 $$f_{kapur} \left( {th} \right) = H_{1}^{c} + H_{2}^{c} , c = \left\{ {\begin{array}{*{20}c} {1,2,3} & {if} & {RGB\;Image} \\ 1 & {if} & {grey\;Image} \\ \end{array} } \right.$$

H1 and H2 are determined by the following formula:

29 $$H_{1}^{C} = \mathop \sum \limits_{i = 1}^{{th_{1} }} \frac{{ph_{i}^{c} }}{{w_{0}^{c} }}\ln \left( {\frac{{ph_{i}^{c} }}{{w_{0}^{c} }}} \right), H_{2}^{C} = \mathop \sum \limits_{i = th + 1}^{L} \frac{{ph_{i}^{c} }}{{w_{1}^{c} }}\ln \left( {\frac{{ph_{i}^{c} }}{{w_{1}^{c} }}} \right)$$

$ph_{i}^{c}$ is the intensity levels’ probability distribution, which can be found using (26).

$w_{0}^{\prime }$ and $w_{i}^{\prime }$ are, respectively, the probability distributions for *C1* and *C2*. The entropy-based strategy, which is similar to Otsu’s method, is extended to include multiple threshold magnitude; in this instance, the image must be divided into *k* classes implementing the same number of thresholds. In such cases, the following is the definition of the new objective function:

30 $$f_{kapur} \left( {TH} \right) = \mathop \sum \limits_{i = 1}^{k} H_{i}^{c} ,c = \left\{ {\begin{array}{*{20}c} {1,2,3} & {if} & {RGB\;{\text{Image}}} \\ 1 & {if} & {grey\;{\text{Image}}} \\ \end{array} } \right.$$

where

31 $$\begin{aligned} H_{1}^{C} & = \mathop \sum \limits_{i = 1}^{{th_{1} }} \frac{{ph_{i}^{c} }}{{w_{0}^{c} }}\ln \left( {\frac{{ph_{i}^{c} }}{{w_{0}^{c} }}} \right) \\ H_{2}^{C} & = \mathop \sum \limits_{{i = th_{1} + 1}}^{{th_{2} }} \frac{{ph_{i}^{c} }}{{w_{1}^{c} }}\ln \left( {\frac{{ph_{i}^{c} }}{{w_{1}^{c} }}} \right) \\ H_{k}^{C} & = \mathop \sum \limits_{{i = th_{k} + 1}}^{L} \frac{{ph_{i}^{c} }}{{w_{k - 1}^{c} }}\ln \left( {\frac{{ph_{i}^{c} }}{{w_{k - 1}^{c} }}} \right) \\ \end{aligned}$$

**Fig. 5:**
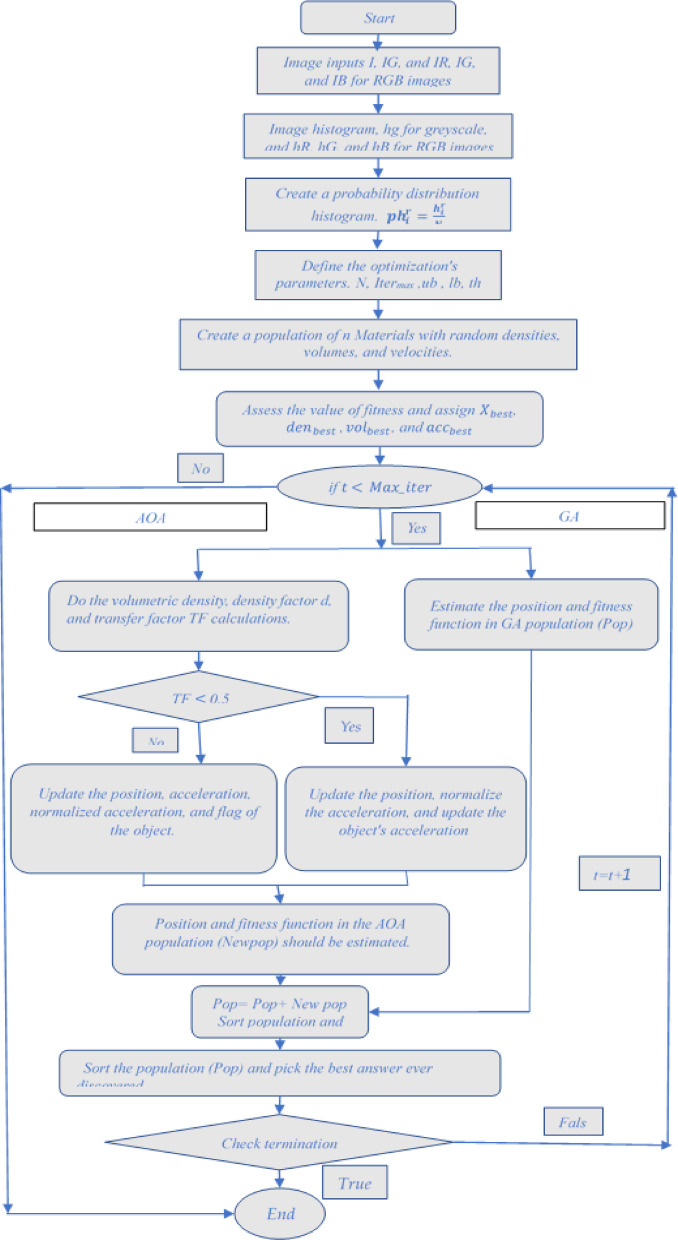
Flowchart of the suggested hybrid GAAOA based image segmentation.

Figure 5 illustrates the image segmentation solution process using the suggested hybrid optimization technique.

## Results and discussions

Two test images are implemented to validate the suggested GAAOA. Camera man and Peppers^45^ are implemented to verify the impact of the suggested hybrid GAAOA technique.

**Fig. 6:**
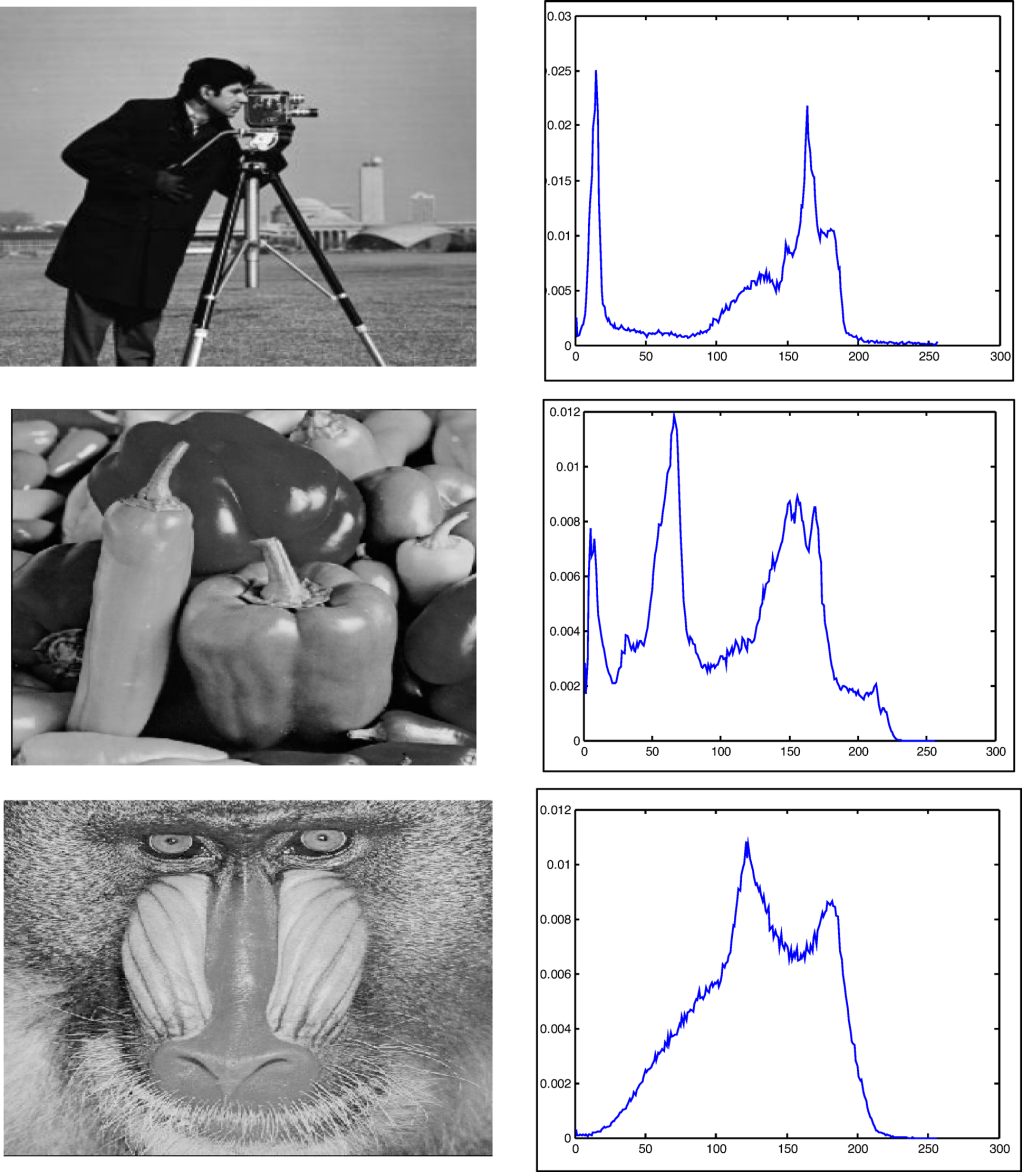
Benchmark test image of camera man, pepper, and baboon and histograms.

Figure 6 shows the histogram distributions for these test images, which describe the number of pixels present at all intensity values. The impact of the suggested GAAOA is compared between algorithm-based multilevel thresholding and other known optimization algorithms such as EMO-Lévy^46^, EMO^45^, CS^42,43^, MFO^16^, WOA^16^, and SCA^44,47^. The identical stop criterion of 100 iterations and 25 populations was used to test all methods. The PSNR is calculated at the conclusion of each test as

32 $$PSNR = 20\log_{10} \left( {\frac{255}{{RMSE}}} \right)$$

The precision of a segmented image in comparison to the original image is measured by the PSNR, which is a significant value.

33 $$RMSE = \sqrt {\frac{{\mathop \sum \nolimits_{i = 1}^{ro} \mathop \sum \nolimits_{j = 1}^{co} \left( {I_{0}^{r} \left( {i,j} \right) - I_{th}^{r} \left( {i,j} \right)} \right)}}{ro \times co}}$$

According to the type of image, ro is the number of rows and co is the number of columns in an image. RGB image r = 1, 2, 3, $I_{th}^{r}$ refers to the segmented image, and $I_{o}^{r}$ is based on the original image.

The stop threshold is at 100 iterations for each trial. At the conclusion of each test, the standard deviation (STD) is calculated (34) in order to confirm the stability. The algorithms become unstable as the STD value rises.

34 $$STD = \sqrt {\mathop \sum \limits_{i = 1}^{{iter_{max} \sum }} \frac{{\left( {s_{i} - m} \right)}}{Ru}}$$

Table 1 identifies the GAAOA parameters. The optimization process’s stop criterion is represented by the upper limit of iterations, which is set at 100. The number of times the accurate fitness values remain unchanged is what is considered the stop criterion, though.

**Table 1:** GAAOA tuning parameters.

The parameters of GA		The parameters of AOA The constant parameters for AOA are as follows:
Crossover probability	0.6, 0.75, or 1	C1 = 2, C2 = 6, C3 = 2, and C4 = 0.5
Mutation probability	0.12, 0.25, 0.35, or 1	
Crossover type	Convex crossover with α = 0.4	
Mutation	Gaussian mutation with σ = 0.01	

Iter local = 100 is the quantity of local searches the algorithm does with a 25-person population each external iteration.

### Test images

The experiments conducted on three benchmark test photos, including Camera Man, Peppers, and Baboon, are utilized to evaluate the efficacy of the optimization technique. The photos are sourced from the USC-SIPI image library, each measuring 512 × 512 pixels^48^. Figure 6 illustrates the test photos with their histogram distributions, which represent the pixel count at each distinct intensity value included in the images. All algorithms were evaluated under identical stopping criteria: 100 iterations and a population of 25.

### Optimal threshold and fitness values

**Fig. 7:**
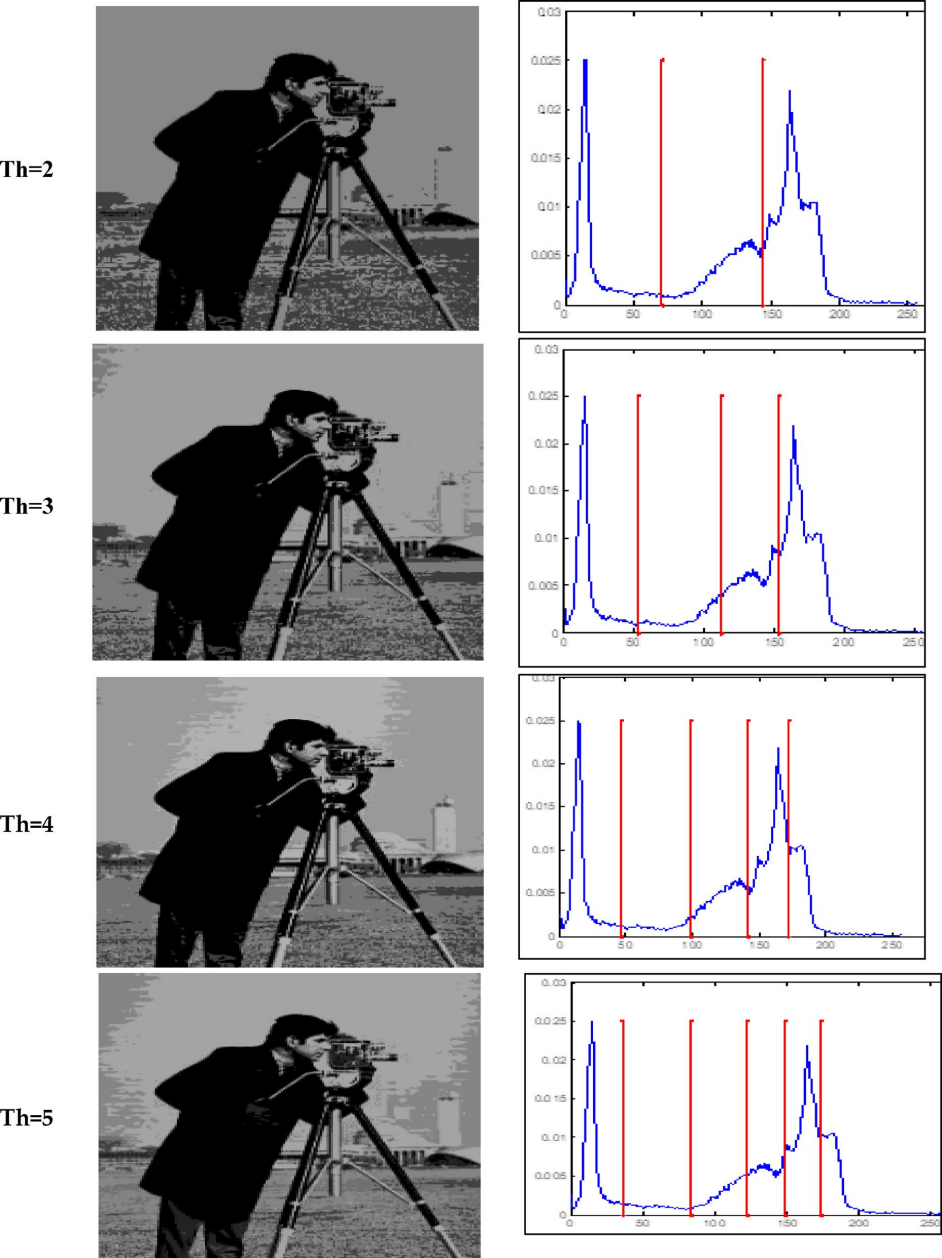
Responses of implementing GAAOA based OTSU’s function.

**Fig. 8:**
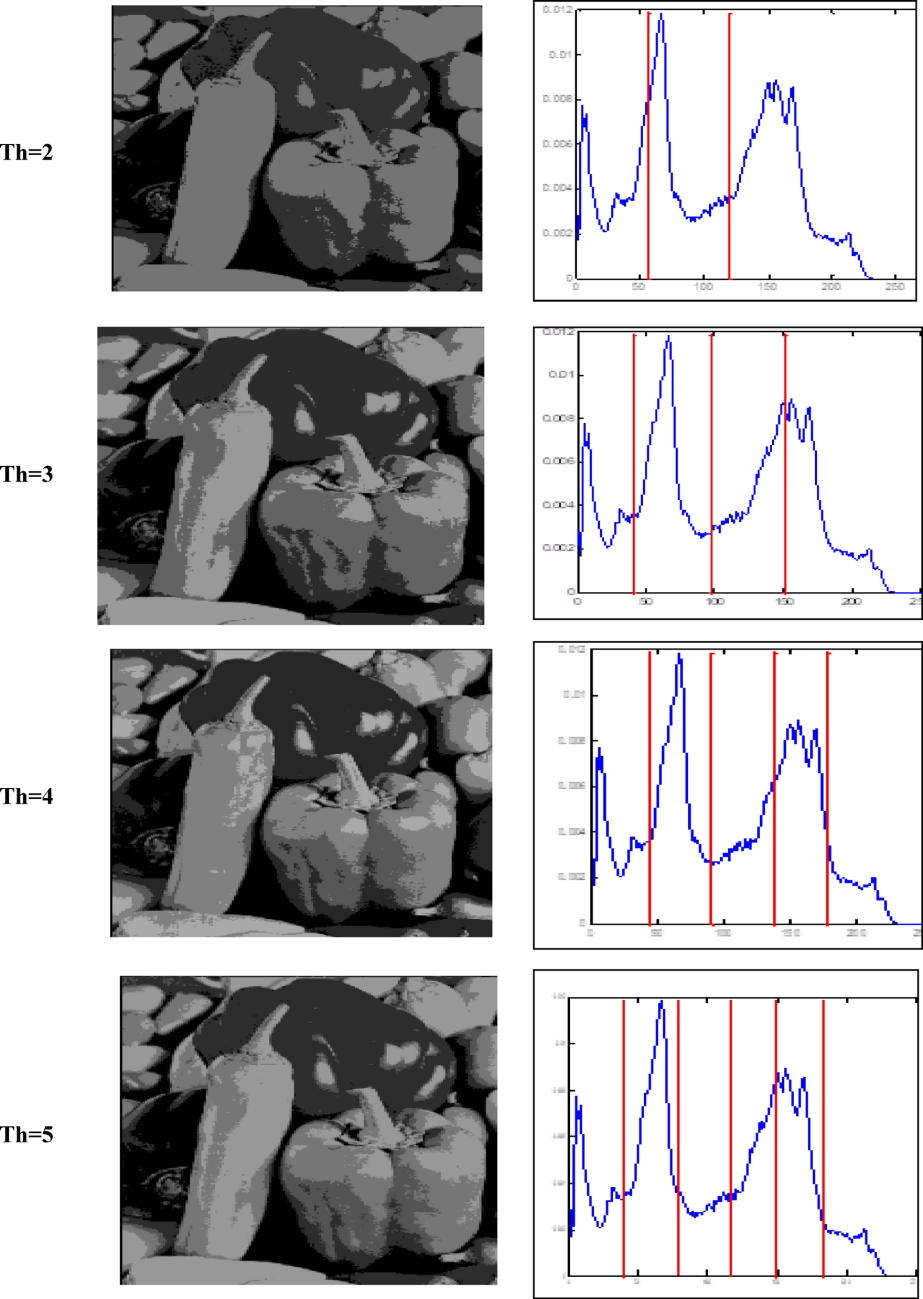
Responses of implementing GAAOA based OTSU’s function.

**Fig. 9:**
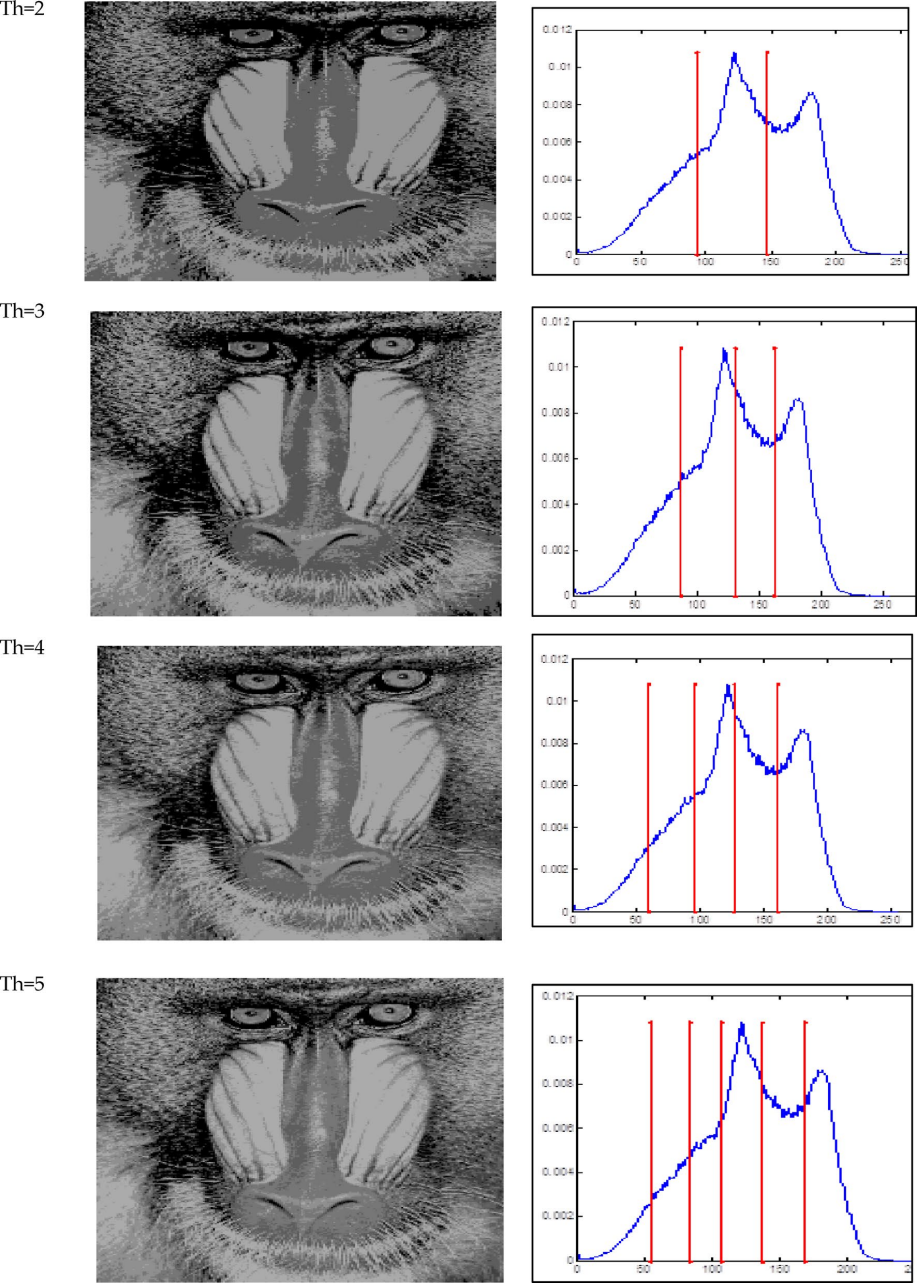
Responses of implementing GAAOA based OTSU’s function.

The Otsu thresholding approach, which is based on the least squares principle, is used in all trials. It is a function of classification category. The entire image pixel will be divided into foreground and background when the value of this classification function reaches its maximum and it becomes clear that threshold k is the best one to utilize for image segmentation. By choosing the ideal threshold, the likelihood of misclassification is reduced and the difference between the foreground and background is greatest. The results of the accurate fitness function and the threshold magnitude for three photos generated by all methods utilizing Otsu’s function are displayed in Tables 2 and 3. Table 3 shows that when TH = 2,3,4,5, the suggested method (GAAOA) outperforms other algorithms in terms of fitness**.** The segmented picture results are shown in Figs. 7, 8 and 9 after applying the four best thresholds (TH = 2,3,4,5) related to Otsu’s objective function.

**Table 2:** The accurate fitness value for different optimization techniques for the test images.

Image	Pepper				Camera man			
	K = 2	K = 3	K = 4	K = 5	K = 2	K = 3	K = 4	K = 5
GAAOA	**2862.6**	**3071.8**	**3187.3**	**3201.3**	**3661.1**	**3743.2**	**3788.1**	**3826.7**
EMO Lévy	2858.5	3059.9	3145.7	3189.8	3646.6	3721.8	3778.2	3809.4
EMO	2857.5	3059.8	3144.8	3189.4	3646.5	3721	3777.4	3809.4
CS	2861.1	3059.9	3145.7	3189.7	3643.6	3718.7	3774.5	3805.7
SCA	2324.5	2550.4	2537.9	2603.8	3515.8	3680.5	3749.18	3718.18
MFO	2435.5	2574.7	2647.67	2669.47	3643.31	3720.25	3748.75	3758.72
WOA	2433.36	2493.18	2632.9	2682.01	3649.36	3690.11	3760.5	3795.86

Image	Baboon							
	K = 2	K = 3	K = 4	K = 5				
GAAOA	1559.7	1551.6	1611.2	1642.1				
EMO Lévy	1558.6	1539.2	1601.4	1628.9				
EMO	1542.5	1532.7	1592.6	1617.2				
CS	1543.9	1539.2	1582.6	1623.6				
SCA	1587.5	1546.3	1562.4	1595.2				
MFO	1621.4	1541.2	1588.2	1610.5				
WOA	1632.3	1530.3	1590.1	1621.4				

Significant values are in bold.

**Table 3:** The best threshold value for different optimization techniques for the test images.

Image	Pepper				Camera man			
	K = 2	K = 3	K = 4	K = 5	K = 2	K = 3	K = 4	K = 5
GAAOA	88.221	47.32	47.67	43.754	74.123	61.431	44.944	39.77
EMO Lévy	82.143	43.99	41.89	39.811	70.144	57.117	41.942	36.83
EMO	56.120	43.10	43.91	39.792	71.144	53.112	47.991	36.83
CS	49.116	43.99	41.89	39.801	70.144	59.119	41.941	37.84
SCA	100.182	92.12	41.139	59.812	96.167	65.155	59.117	47.85
MFO	56.152	72.11	47.74	29.859	94.146	59.143	77.135	37.53
WOA	66.143	59.12	68.94	38.812	86.145	73.151	76.126	80.12

Image	Baboon							
	K = 2	K = 3	K = 4	K = 5				
GAAOA	99.12	89.34	76.93	67.76				
EMO Lévy	96.98	88.09	71.23	64.95				
EMO	94.91	81.21	68.56	62.34				
CS	88.03	76.82	71.23	68.76				
SCA	78.34	69.86	45.65	75.67				
MFO	70.45	64.37	44.8	46.98				
WOA	68.2	60.32	96.08	32.77

**Fig. 10:**
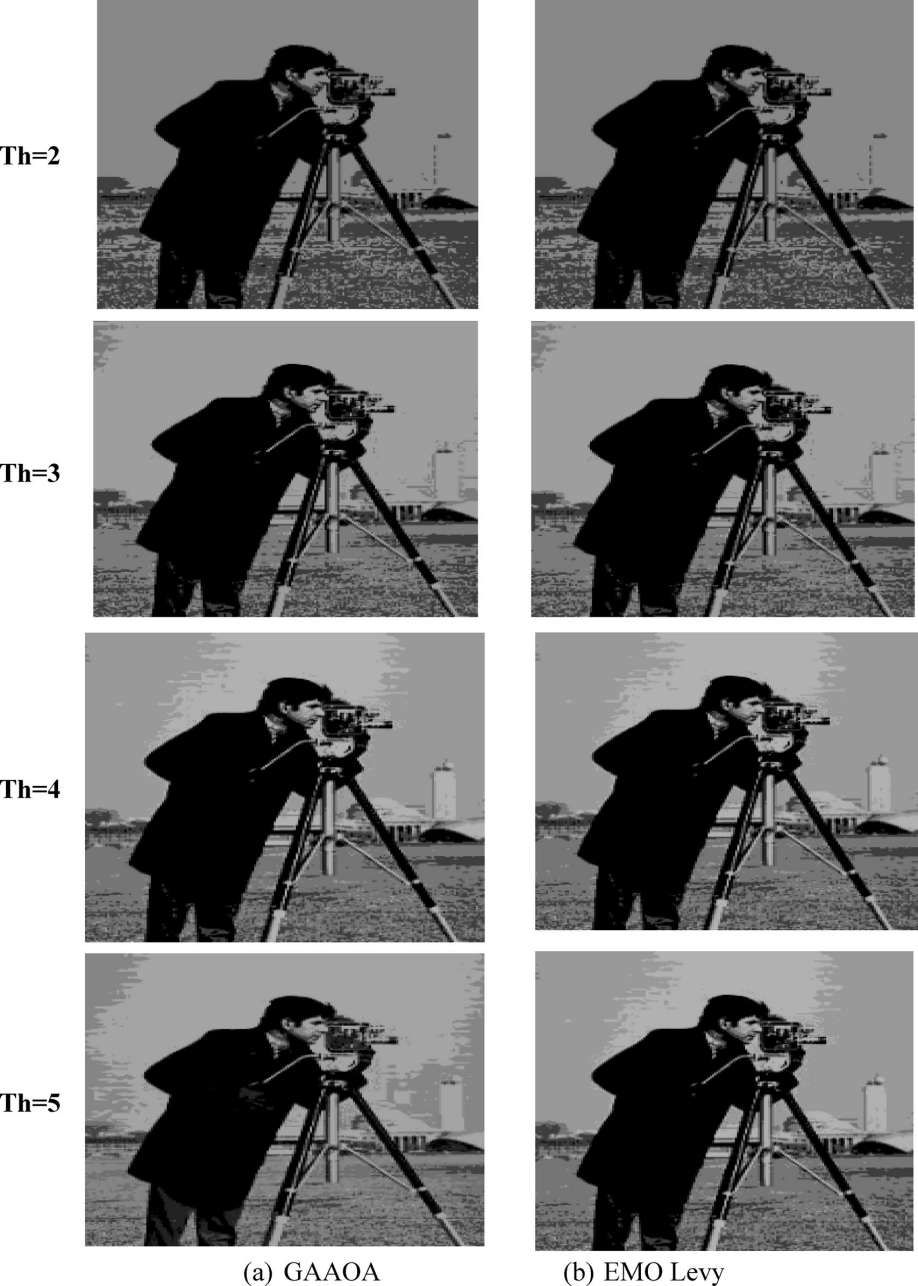
A Segmentation benchmark image after apply GAAOA and IMEMO algorithm.

**Fig. 11:**
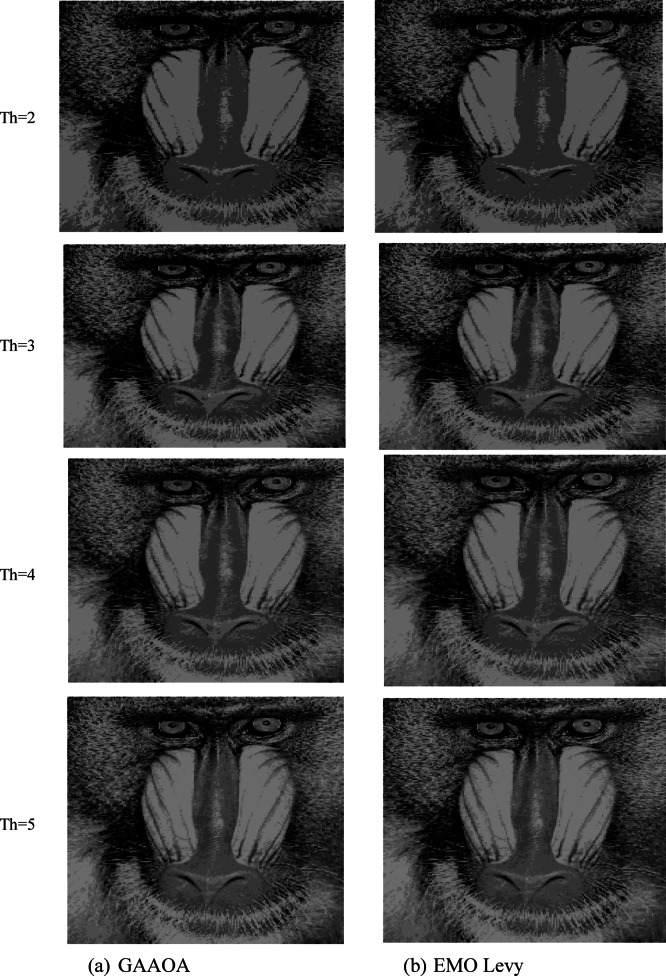
A Segmentation benchmark image after apply GAAOA and IMEMO algorithm.

The segmentation results of the suggested algorithms and the other algorithms with various threshold values (TH = 2,3,4,5) are displayed in Figs. 10 and 11. These numbers allow us to draw the conclusion that higher-level images have more details than lower-level images.

Table 4 demonstrates that when weighed against additional multilayer algorithms (GAAOA, CS, MFO, WOA, SCA), the proposed method typically has the highest values for PSNR. Also take note that the PSNR value for all algorithms grows any time the number of threshold magnitudes does. When comparing a segment image’s performance and accuracy to the original image, the PSNR is a crucial metric to consider.

**Table 4:** The average value of PSNR measure of different optimization algorithms.

Image	Pepper				Camera man			
	K = 2	K = 3	K = 4	K = 5	K = 2	K = 3	K = 4	K = 5
GAAOA	17.783	19.123	21.126	22.201	19.328	21.712	22.165	24.513
EMO Lévy	16.4296	18.8469	20.4749	21.8597	17.3213	20.2304	21.4177	23.2749
EMO	15.5245	18.8675	20.4503	21.8329	17.253	20.2116	21.625	23.2749
CS	16.2354	18.8675	20.4503	21.855	17.253	20.1796	21.4177	23.2735
SCA	16.6278	18.1623	20.63	21.4056	17.3744	20.0161	21.9564	21.6302
MFO	16.2716	18.0953	19.7982	21.608	18.2862	19.9084	21.1897	22.1592
WOA	14.2092	16.466	16.9392	19.5709	15.8362	18.0557	20.219	18.6505

Image	Baboon							
	K = 2	K = 3	K = 4	K = 5				
GAAOA	16.943	17.653	19.126	21.387				
EMO Lévy	16. 296	18.8469	21.4749	22.8597				
EMO	15.524	17.675	19.4503	22.875				
CS	16.654	17.879	20.324	22.855				
SCA	16.827	18.654	21.432	23.4056				
MFO	15.783	17.0953	20.7982	22.762				
WOA	15.734	17.832	18.367	20.321				

The CPU time for all techniques when used to solve the multilevel thresholding problem is shown in Table 5. On a computer with an Intel(R) Core(TM) i7-4770 CPU i5 processor running at 3.40GHz and 16 GB of RAM, experiments were carried out using MATLAB R2014. The created technique uses less CPU time than the conventional EMO and CS algorithm, as can be seen from the table.

**Table 5:** The executing time of different optimization algorithms.

Image	Pepper				Camera man			
	K = 2	K = 3	K = 4	K = 5	K = 2	K = 3	K = 4	K = 5
GAAOA	8.23	10.12	11.32	11.54	4.43	7.12	10.12	14.51
EMO Lévy	9.67	12.16	14.78	16.38	5.34	8.90	12.29	16.26
EMO	10.31	14.47	16.26	18.43	7.53	10.95	14.39	18.29
CS	12.31	15.48	17.45	19.85	10.32	12.36	16.23	19.43
SCA	13.95	15.78	18.66	20.28	11.03	13.02	17.15	20.33
MFO	14.33	16.09	19.23	21.91	12.22	13.89	17.87	20.85
WOA	14.87	16.59	20.01	22.01	12.83	14.66	18.32	21.22

Image	Baboon							
	K = 2	K = 3	K = 4	K = 5				
GAAOA	3.45	5.34	8.43	10.46				
EMO Lévy	3.95	6.32	9.34	11.56				
EMO	4.36	6.12	8.34	10.93				
CS	5.23	6.34	7.98	9.93				
SCA	4.89	5.89	7.49	10.34				
MFO	4.74	4.98	6.79	9.65				
WOA	4.23	6.34	8.96	11.24				

In Table 6, the two multilevel segmentations based on thresholding techniques Otsu and Kapur are contrasted. This table shows that, in comparison to the Kapur approach, the Otsu thresholding approach produces segmented images with great precision and maximum magnitude of PSNR and STD.

**Table 6:** Comparative study between fitness value, thresholds, and PSNR for low-contrast image (Camera man) for different optimization techniques.

Image	Fitness value				Thresholds				PSNR			
	K = 2	K = 3	K = 4	K = 5	K = 2	K = 3	K = 4	K = 5	K = 2	K = 3	K = 4	K = 5
GAAOA Lévy	1.15e+3	1.181e+3	1.21e+3	1.26e+3	53.92	37.22	32.23	34.23	22.61	25.21	26.11	28.12
EMO Lévy	1.15e+3	1.18e+3	1.199e+3	1.23e+3	52.95	35.68.	31.66	29.53	21.48	24.56	25.62	27.31
EMO	1.15e+3	1.18e+3	1.191e+3	1.21e+3	52.94	34.69.	34.68	33.67	21.34	24.43	25.55	25.78
CS	1.15e+3	1.18e+03	1.189e+3	1.12e+3	52.93	34.67	34.68	32.65	21.48	24.56	25.62	26.84
SCA	1.14e+3	1.17e+3	1.184e+3	1.119e+3	52.34	34.13	34.21	32.28	21.16	24.23	25.41	26.23
MFO	1.13e+3	1.169e+3	1.183e+3	1.114e+3	52.22	34.01	33.97	32.11	21.02	24.12	25.22	26.12
WOA	1.13e+3	1.167e+3	a.a81e+3	1.111 e+3	51.87	33.87	33.67	32.01	20.97	23.89	25.12	25.76

### Minimum and maximum contrast test image

**Fig. 12:**
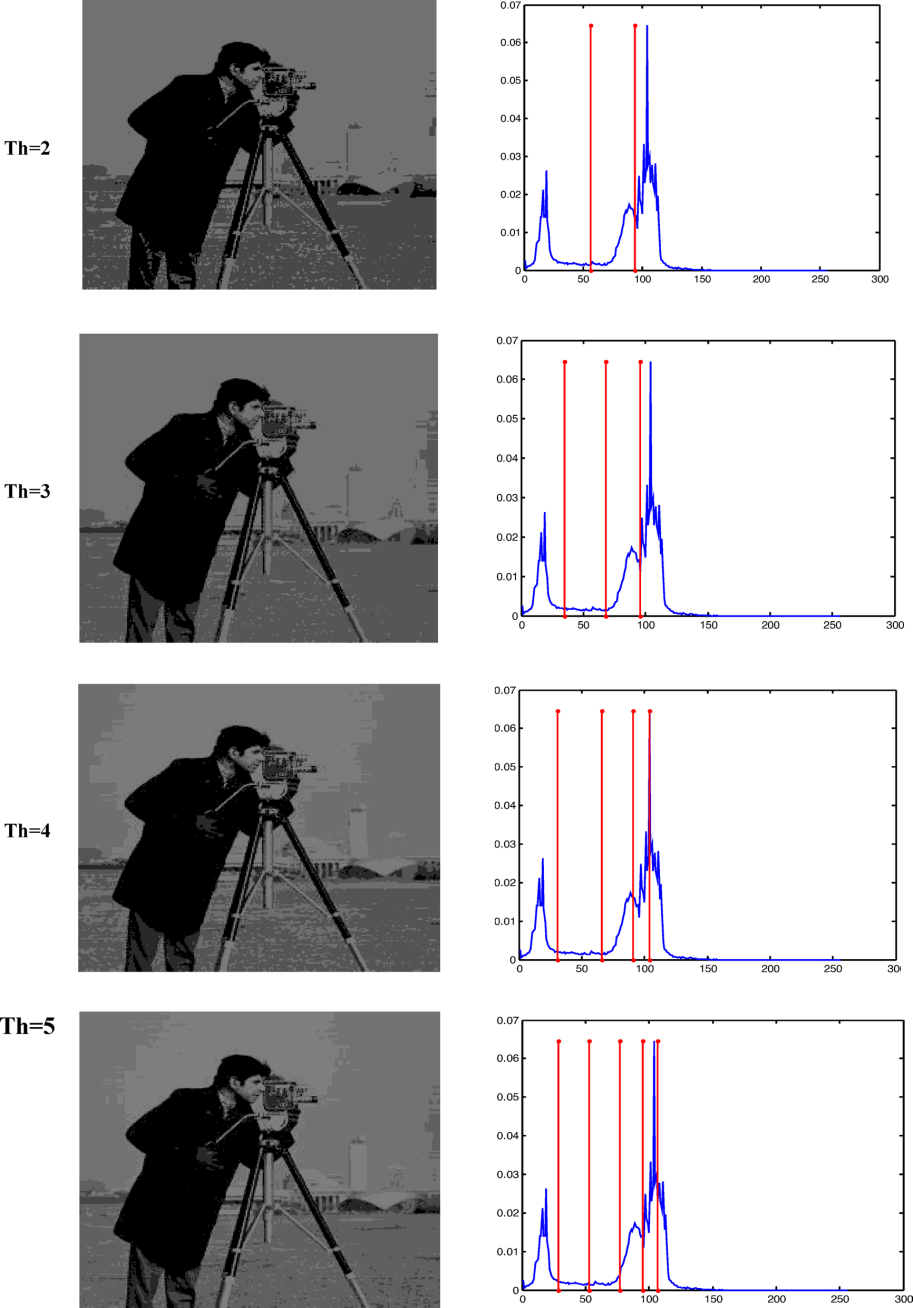
The suggested optimization technique for low contrast images.

**Fig. 13:**
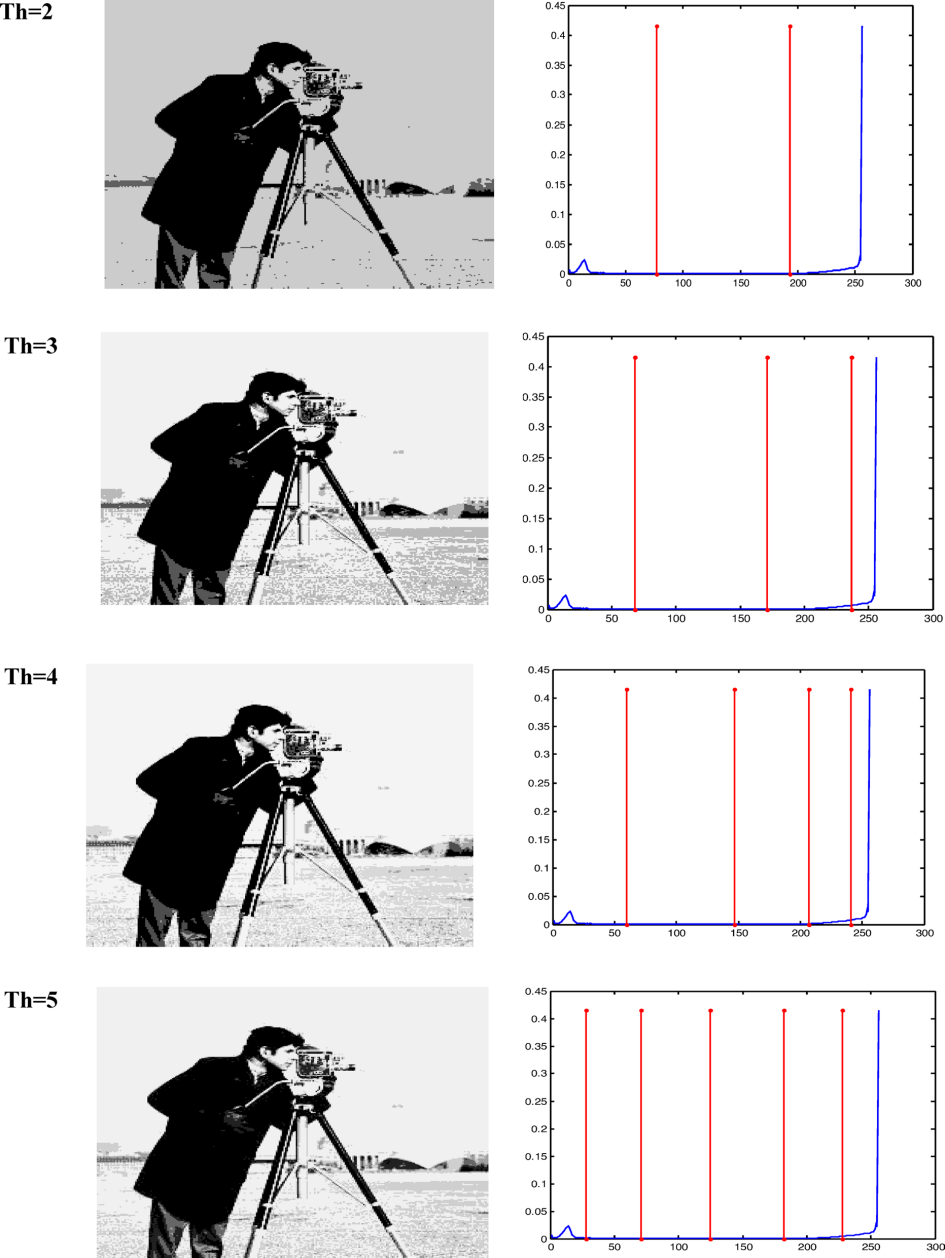
The suggested optimization technique for high contrast images.

To indicate the impact of the suggested GAAOA technique, low- and high-contrast are implemented to camera man image for different threshold values (Th = 2, 3, 4, 5). Figure 12 shows the application of the suggested hybrid GAAOA for low-contrast image. Figure 13 depicts the implementation of the suggested GAAOA for high-contrast image. Table 6 shows the comparative study between fitness value, thresholds, and PSNR for low-contrast image (Camera man) for different optimization techniques and the suggested GAAOA technique in low-contrast image. The results show the accuracy of the suggested technique in comparison with the published technique. Table 7 shows the comparative study between fitness value, thresholds, and PSNR for low-contrast image (Camera man) for different optimization techniques and the suggested GAAOA technique. The results show the accuracy of the suggested technique in comparison with the published technique in high-contrast image. Also, we concluded that the suggested hybrid GAAOA has better performance in comparison with other techniques in terms of threshold, PSNR, and fitness value.

**Table 7:** Comparative study between fitness value, thresholds, and PSNR for high-contrast image (Camera man) for different optimization techniques.

Image	Fitness value				Thresholds				PSNR			
	K = 2	K = 3	K = 4	K = 5	K = 2	K = 3	K = 4	K = 5	K = 2	K = 3	K = 4	K = 5
GAAOA	10.3e+3	9.63+3	9.7e+3	9.6e+3	79.11	71.12	65.11	31.23	19.78	21.27	23.76	24.12
EMO Lévy	9.2e+3	9.4e+3	9.4e+3	9.4e+3	77.2	68.17	60.21	28.71	18.93	19.62	22.38	22.79
EMO	9.2e+3	9.3e+3	9.4e+03	9.4e+3	78.2	68.17	42.11	39.10	13.95	19.57	21.37	22.04
CS	8.6e+3	8.76e+3	8.7e+3	8.7e+3	73.2	66.16	41.11	37.93	12.86	19.21	20.82	22.43
SCA	8.61e+3	8.72e+3	8.69e+3	8.66e+3	73.11	65.97	40.91	36.67	12.78	18.81	20.77	21.98
MFO	8.43e+3	8.71e+	8.67e+3	8.62e+3	72.29	65.45	40.12	36.44	12.71	18.76	20.71	21.82
WOA	8.44e+3	8.701e+	8.63e+3	8.61e+3	72.16	65.34	39.23	36.21	12.65	18.21	20.13	21.65

According to the results, the suggested algorithm has a strong computing capability, outperforms, and converges quickly while using low time and less energy. The suggested algorithm’s threshold and fitness function values are considered a disadvantage since they are frequently not optimal for the instances under study.

### Code availability

The custom code used in this study is part of ongoing research and is currently not publicly available. However, it can be provided by the authors upon reasonable request for academic and non-commercial use. Interested readers may contact the corresponding author to obtain access.

## Advantages and drawbacks of the suggested hybrid GAAOA

According to the responses, the proposed algorithm, which is shown in Table 4, provides the maximum PSNR values in the majority of the analyzed scenarios when compared to other algorithms.

When TH = 2, 3, 4, 5, as shown in Table 2, the suggested method (GAAOA) provides the accurate fitness magnitudes in the majority of cases compared to the other optimization techniques.

As depicted in Table 5, the computation time of the suggested algorithm is shorter than that of the results obtained by the other optimization strategies.

The suggested algorithm’s dependability has been demonstrated utilizing a variety of high- and low-contrast images.

## Conclusions

This work proposed a hybrid GAAOA combining Genetic Algorithm (GA), Archimedes Optimization Algorithm (AOA), and Lévy flight to enhance multilevel thresholding for image segmentation. The method was evaluated on standard benchmark images and demonstrated superior performance over state-of-the-art and recent hybrid optimizers like AOA-HHO, HADECO, and CVWOA, achieving higher PSNR, faster convergence, and lower computation time.22, 

The improved results stem from the synergy between GA crossover (enhancing local search) and Lévy flight (enhancing global exploration). Although this paper does not include ablation analysis due to space constraints, we are currently conducting such studies to assess the individual contributions of each component, with results to be published separately.

Advantages and limitations of the method are outlined in Section “Advantages and drawbacks of the suggested hybrid GAAOA”. While GAAOA performs robustly across various test cases, its effectiveness may vary in real-time or highly textured scenarios, which are proposed for future research.

Finally, the method holds strong potential for practical applications in medical imaging, industrial inspection, and remote sensing, with planned future extensions including real-time processing and integration with deep learning.

## Data Availability

The authors confirm that the data supporting the findings of this study are available within online in https://sipi.usc.edu/database/.
